# Traditional Uses, Phytochemistry, and Pharmacological Activities of *Vernonia cinerea* (L.) Less.: An Updated Review

**DOI:** 10.3390/nu16091396

**Published:** 2024-05-06

**Authors:** Nguyen Minh Trang, Le Ba Vinh, Nguyen Viet Phong, Seo Young Yang

**Affiliations:** 1 College of Pharmacy, Chungnam National University, Daejeon 34134, Republic of Korea; ngminhtrang52@gmail.com; 2 Institute of Marine Biochemistry (IMBC), Vietnam Academy of Science and Technology (VAST), 18 Hoang Quoc Viet, Hanoi 10072, Vietnam; vinhrooney@gmail.com; 3 Department of Biology Education, Teachers College and Institute for Phylogenomics and Evolution, Kyungpook National University, Daegu 41566, Republic of Korea

**Keywords:** *Vernonia cinerea* (L.) Less., botanical characterization, plant distribution, traditional uses, phytochemistry, pharmacological activities

## Abstract

*Vernonia cinerea* (L.) Less. is a perennial herbaceous plant found mainly in tropical areas, particularly in Southeast Asia, South America, and India. Various parts of *V. cinerea* have traditionally been used in folk medicine to treat several diseases, such as malaria, fever, and liver diseases. *V. cinerea* has so far yielded about 92 secondary metabolites. The majority of these are sesquiterpene lactones, but triterpenes, flavonoids, steroids, phenolics, and other compounds are present as well. *V. cinerea* crude extracts reportedly exhibit anti-inflammatory, antiprotozoal, antidiabetic, anticancer, antimicrobial, antioxidant, and renoprotective activities. This study aims to provide the latest up-to-date information on the botanical characterization, distribution, traditional uses, phytochemistry, and pharmacological activity of *V. cinerea*. Information on *V. cinerea* was thoroughly reviewed. The literature published between 1950 and 2024 was compiled through online bibliographic databases, including SciFinder, Web of Science, Google Scholar, PubMed, ScienceDirect, Springer Link, Wiley, and the MDPI online library. The keywords used for the literature search included *Vernonia cinerea* (L.) Less. and the synonyms *Cyanthillium cinereum* (L.) H.Rob., *Conyza cinerea* L., and various others.

## 1. Introduction

*Vernonia cinerea* (L.) Less. has the synonym *Cyanthillium cinereum* (L.) H.Rob. and is usually known as “Sahadevi” in Indian or “Bach dau ong” in Vietnamese traditional medicine [1,2]. *Vernonia*, a genus within the Asteraceae family, contains the largest number of species in the *Vernoniae* tribe, with around 1000 species [3]. The genus was named after William Vernon, a botanist who first identified and classified this diverse group of plants in the late 1600s [4]. *V. cinerea* is distributed mainly in tropical regions, from Asia to Africa to Australia [5]. In Ayurveda, *V. cinerea* has long been used as a traditional therapy to treat eruptive boils, worms, skin diseases, leprosy, and arthritis [6,7]. This plant is also used in Vietnam as a natural sedative and analgesic [2]. Because of its extensive traditional uses, scientific communities have invested significant effort into *V. cinerea*, including numerous investigations into its bioactive metabolites and pharmacological effects. More than 92 compounds, particularly sesquiterpene lactones, have been reportedly isolated and structurally elucidated from *V. cinerea*. Pharmacological research has shown that *V. cinerea* exhibits diverse biological effects, including antioxidant, anti-inflammatory, antitumor, and antidiabetic properties, as well as an efficacy in aiding smoking cessation [8,9,10,11,12]. Although several reviews concerning the ethnopharmacological and biological activities of *V. cinerea* have been published [5,13,14], none of them comprehensively cover all the details and recently discovered aspects of this plant. Thus, this review aims to provide an updated and thorough overview of the botany, traditional applications, secondary metabolites, chemical profiling, and pharmacological activities of this plant. We hope this review will contribute to a more comprehensive understanding of the potential of the bioactive compounds found in this medicinal plant, thus highlighting its promise as a candidate for future medicinal use.

## 2. Taxonomic Position

Kingdom—Plantae

Phylum—Tracheophyta

Class—Magnoliopsida

Order—Asterales

Family—Asteraceae

Genus—Vernonia

Species—*Vernonia cinerea* (L.) Less.

## 3. Botanical Characterization and Distribution

**Figure 1:**
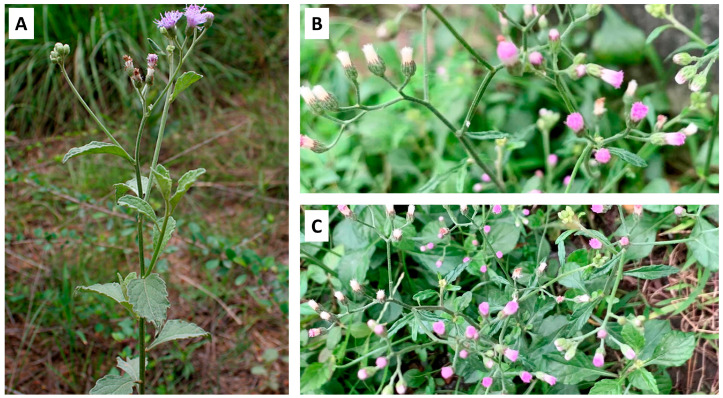
*Vernonia cinerea*. The (**A**) natural habitat and (**B**,**C**) flowers of *V. cinerea*.

*Vernonia cinerea* is a branching, erect, or infrequently decumbent plant that grows to a height of 12–75 cm (Figure 1A). The roots consist of a main root that is 5–12 cm long and 1–7 mm thick, as well as gradually tapering, oblique, and bearing few rootlets. The external surface of the roots is filthy brown, with short fractures. The leaves are simple, dark green, smooth, alternate, exstipulate, 2.5–5 cm long, and 1.8–3.6 cm wide. The upper leaves are tiny, linear, and irregularly toothed, whereas the lower leaves are oval with whole or subentire edges. The petioles are winged and short. The stem is erect, thin, glabrous, and slightly branched, with a length of 10–17 cm and a thickness of 1–8 mm. The inflorescence is a flat-topped panicle with many capitula composed of purplish or pinkish disc florets, each approximately 3 mm in diameter on slender pedicels (Figure 1B,C) [1,2].

*V. cinerea* is primarily found along roadsides and in open waste areas, dry grassy sites, and perennial crop plantations. It is widely distributed in tropical regions worldwide, including Central and South America, Africa, the Middle East, China, Southeast Asia, and Australia [15,16,17].

## 4. Ethnopharmacological and Traditional Uses

In its ethnobotanical applications, *V. cinerea* is one of the most widely used species of the *Vernonia* genus, with a long history of traditional usage in Ayurveda, traditional Asian medicine, and Western herbalism [3].

### 4.1. India

Ayurveda, the science of life, was developed about 6000 years ago in India. In the history of the world, it is the earliest record of scientific medicine [18]. In the *Ayurvedic Pharmacopoeia of India*, *V. cinerea* is commonly called “Sahadevi”. Sahadevi has been widely utilized for collyrium preparation since ancient times. Interestingly, eye salve is currently made from the essence of this plant and is regarded as the best medication for treating most eye problems [19]. According to the *Ayurvedic Pharmacopoeia of India*, this herb is also used to treat eruptive boils, worm and skin diseases, leprosy, arthritis, and conjunctivitis [6,20,21]. In India, the leaf of this plant is widely used to treat colds, fevers, and especially malaria [7,22].

Furthermore, the leaves are also considered to cure some bacterial illnesses such as leprosy and scabies [23], and the whole plant is effective in the treatment of hemorrhoids and fever [24]. Recently, this plant has been investigated as a potential therapeutic agent for lung cancer therapy [25,26].

### 4.2. Other Asian Countries

In several South Asia nations, such as Nepal and Sri Lanka, *V. cinerea* leaves are used to treat inflammation, wounds, liver function issues, cough, asthma, bronchitis, and gastrointestinal diseases [27,28]. In Malaysia, the juice of the finely pounded root and leaf is drunk to treat cancer and asthma [29,30]. In Thailand, this herb is used to treat fever, wounds, diabetes, diarrhea, muscle pain, and gastrointestinal healing [31,32]. In Vietnam, *V. cinerea* is crushed into small pieces, dried, and used as a natural sedative and analgesic.

### 4.3. African Countries

The leaves of *V. cinerea* are traditionally used in the treatment of vermifuge, tonsillitis, and fever [33,34,35]. Interestingly, the stem of this plant is considered a good luck charm in Uganda [36].

### 4.4. West Indies Countries

*V. cinerea* leaves are used as a medication for measles treatment in some West Indies countries [37].

## 5. Phytochemistry

**Figure 2:**
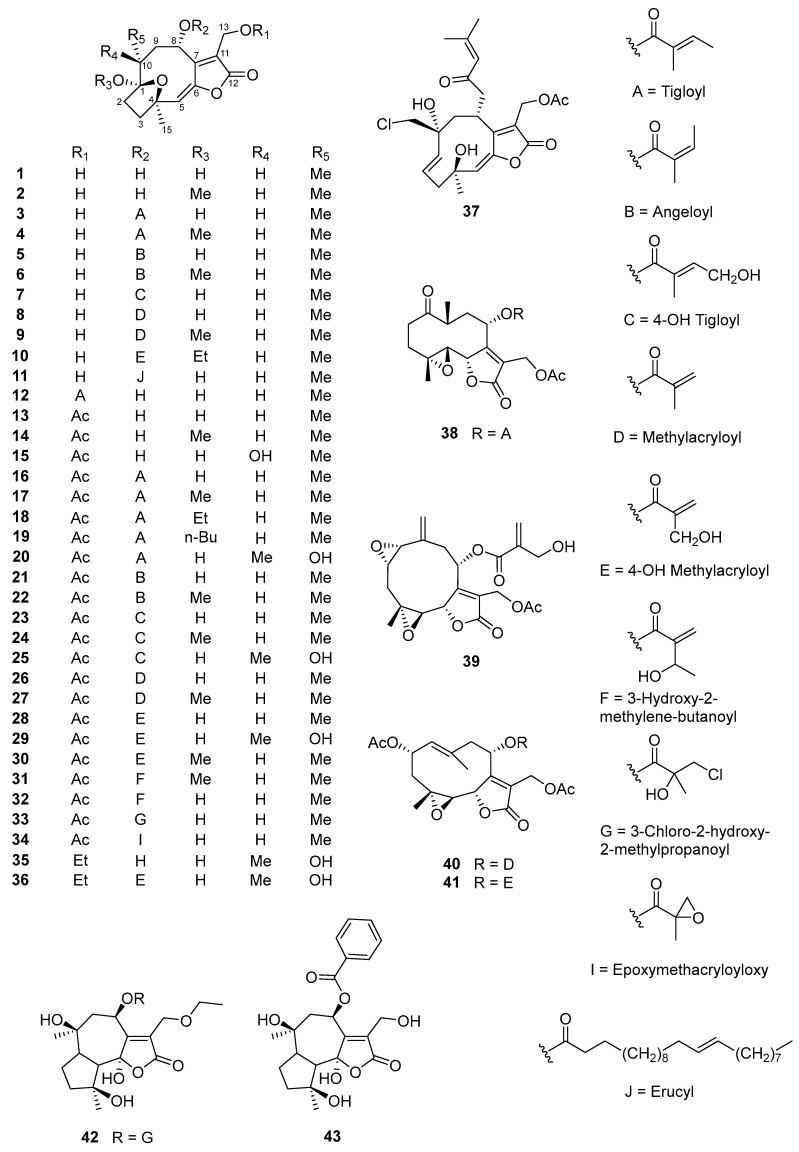

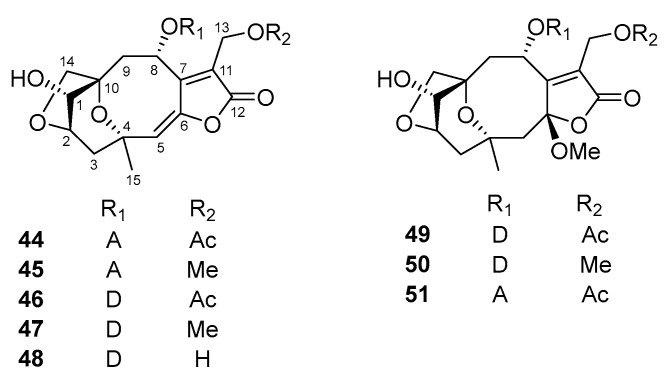
Chemical structures of sesquiterpene lactones (**1**–**51**) from *V. cinerea*.

**Figure 3:**
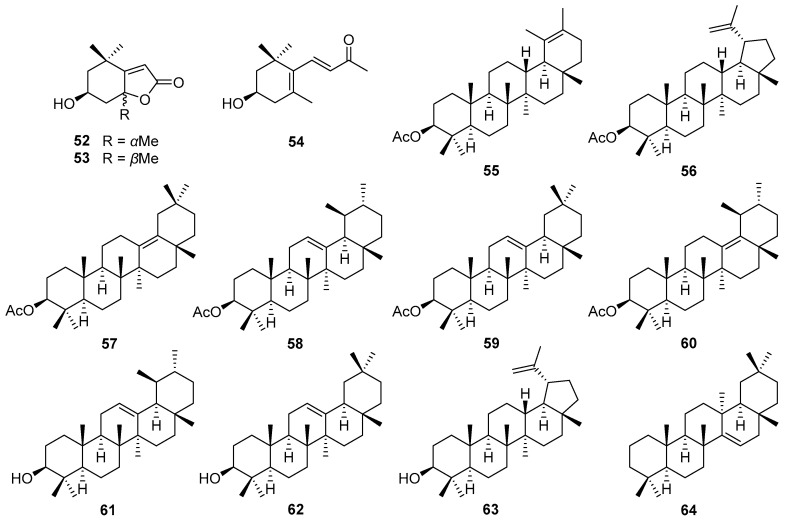
Chemical structures of norisoprenoids (**52**–**54**) and triterpenes (**55**–**64**) isolated from *V. cinerea*.

**Figure 4:**
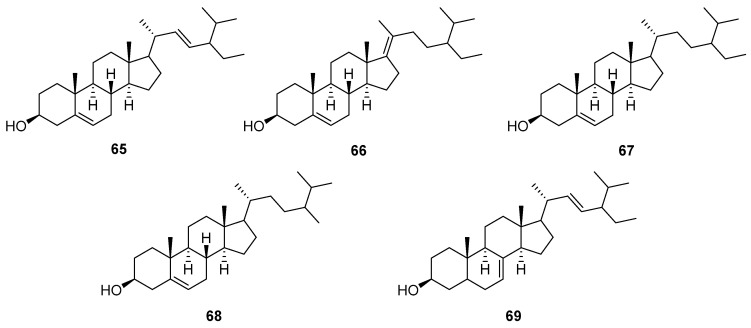
Chemical structures of steroids (**65**–**69**) isolated from *V. cinerea*.

**Figure 5:**
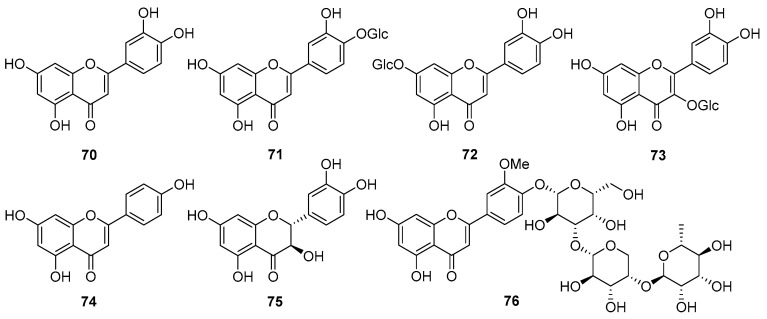
Chemical structures of flavonoids (**70**–**76**) isolated from *V. cinerea*. The abbreviation “Glc” in **71**–**73** denotes *β*-D-glucopyranosyl.

**Figure 6:**
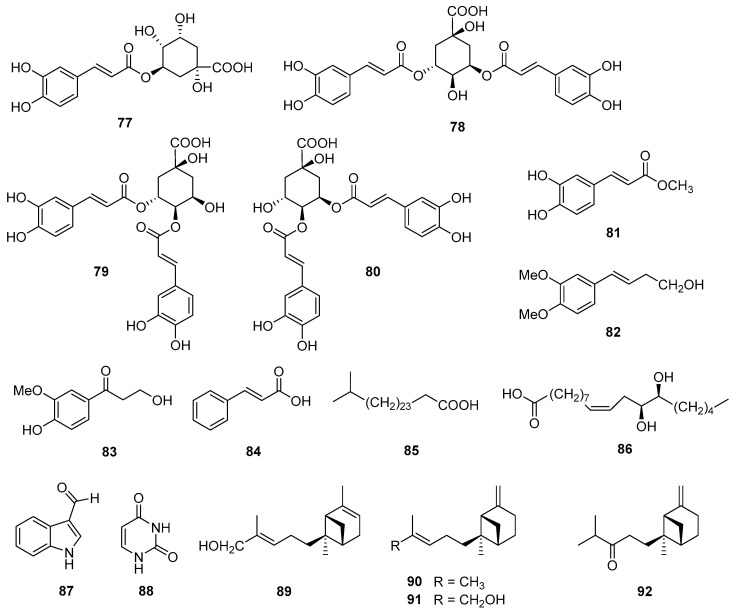
Chemical structures of phenolic compounds (**77**–**84**) and other compounds (**85**–**92**) isolated from *V. cinerea*.

A detailed literature study revealed that terpenoids, especially sesquiterpene lactones, are major secondary constituents of *V. cinerea*, with a variety of terpenoids, such as C11-terpene lactone, megastigmane, and triterpene, being identified. In addition, steroids, flavonoids, phenolics, and other compounds have reportedly been isolated using different extraction techniques and from different plant parts of *V. cinerea* (i.e., roots, flowers, stems, and leaves). In total, about 92 chemical constituents have thus far been isolated and structurally characterized. The isolated compounds are listed in Table 1**,** and their chemical structures are illustrated in Figure 2, Figure 3, Figure 4, Figure 5 and Figure 6. The compounds are numbered consecutively, **1**–**92**, in the table and figures. These numbers are consistent between Table 1 and Figure 2, Figure 3, Figure 4, Figure 5 and Figure 6 and often used to identify them in the text.

### 5.1. Sesquiterpene Lactones

Sesquiterpene lactones are a diverse group of terpenoids (15-carbon compounds) with a characteristic isoprenoid ring system: a lactone ring containing a conjugated exomethylene group (*α*-methylene-*γ*-lactone) [38,39]. Many of the major bioactive secondary metabolites of *V. cinerea* are sesquiterpene lactone derivatives. To date, a total of 51 sesquiterpene lactones have been identified. In this review, all sesquiterpene lactones of *V. cinerea* from the literature (**1**–**51**) have been cataloged and are shown in Figure 2.

In 1986, Jakupovic et al. [40] reported the first isolation of sesquiterpene-lactone-type constituents. Four new sesquiterpene lactones named 8*α*-tigloyloxyhirsutinolide-13-*O*-acetate (**16**), 8*α*-(4-hydroxytigloyloxy)-hirsutinolide-13-*O*-acetate (**23**), 8*α*-(4-hydroxytigloxyloxy)-10*α*-hydroxyhirsutinolide-13-*O*-acetate (**25**), and 8*α*-(4-hydroxymethacryloyloxy)-10*α*-hydroxyhirsutinolide-13-*O*-acetate (**29**), together with two known compounds, 8*α*-(4-hydroxymethacryloyloxy)-hirsutinolide-13-*O*-acetate (**28**) and stilpnotomentolide-8-*O*-tiglate (**38**), were isolated from the aerial parts of *V. cinerea* collected in Costa Rica. In addition, a collection from Malawi yielded glaucolide E (**40**) and two new lactones, a hydroxymethacrylate of glaucolide E—19-hydroxyglaucolide E (**41**)—and a bisepoxide named vernocinerolide-8-*O*-(4-hydroxymethacrylate) (**39**). These results suggest that the phytochemicals of the plant from the two localities were clearly different. However, the isolated compounds are all related to the precursors of the glaucolides, although the compounds from the material plant collected in Malawi are less common in *Vernonia* species [40]. These results also show that the synthesis and accumulation of secondary metabolites in medicinal plants are extremely complicated, with multiple factors influencing it, including genetic circuits—such as controlled genes and enzymes—and, in particular, environmental characteristics like location, light, temperature, water, air pressure, etc. [41]. Other hirsutinolide-type sesquiterpene lactones, which possess an *α,β*-unsaturated-*γ*-lactone ring and a 1*β*,4*β*-ether ring as functional groups, named vernolide-A (**4**) and -B (**15**), were isolated from the ethanolic extracts of *V. cinerea* stems by Kuo et al. in 2003 [42]. In 2012, Youn et al. [15] published the first study on the secondary metabolites of *V. cinerea* flowers. A new sesquiterpene lactone named 8*α*-hydroxyhirsutinolide (**1**) and its derivative, 8*α*-hydroxyl-1-*O*-methylhirsutinolide (**2**), along with 8*α*-tigloyloxyhirsutinolide (**3**), hirsutinolide-13-*O*-acetate (**13**), 8*α*-(2-methylacryloyloxy)-hirsutinolide-13-*O*-acetate (**26**), and 8*α*-(2-methylacryloyloxy)-1*α*-methoxyhirsutinolide-13-*O*-acetate (**27**), were isolated from a hexane extract of *V. cinerea* flowers and structurally elucidated. Furthermore, based on NOESY and X-ray crystallographic analysis, the relative stereochemistry of compound **3** was characterized, with H-10*β* and H-9*β* located on the same side as the C-2–C-3 bridge and the 1,4-epoxy ring located below the ten membered ring [15].

In 2019, Zhang et al. [43] described the isolation of eight new sesquiterpene lactones, named vercinolides A–H (**44**–**51**), which were isolated from whole *V. cinerea* plants collected in Lampang province, Thailand. Their relative configurations were confirmed through high-resolution electrospray ionization mass spectrometry (HR-ESI-MS) and NOESY, and by using Mosher experiments and electronic circular dichroism (ECD) analysis, their absolute configurations were finally determined. Notably, the new compounds (**44**–**51**) were the first members of a novel class of sesquiterpene lactones possessing a rare 4*α*,10*α*-ether ring and a 2,14-ether ring. Recently, Ang et al. [44] reported the isolation of four new hirsutinolide-type sesquiterpenoids, named cyanolides A–D (**37**, **18**, **19**, and **10**), from the aerial parts of the plant. Cyanolide A (**37**) is a hirsutinolide sesquiterpenoid with several particularly rare characteristics: a ruptured 1,4-ether ring and the presence of a chlorine atom.

### 5.2. Norisoprenoids and Triterpenes

In addition to terpenoids, norisoprenoids and triterpenes are also major constituents of *V. cinerea*. The structures of three norisoprenoids (**52**–**54**) and ten triterpenes (**55**–**64**) are shown in Figure 3. In 2014, Youn et al. [45] reported the first isolation of norisoprenoid-type metabolites—two C11-norisoprenoids, including loliolide (**52**) and isololiolide (**53**), along with a C13-norisoprenoid, (3*R*)-3-hydroxyionone (**54**)—from the methanol extract of *V. cinerea* leaves and stems.

Triterpenes are a group of phytochemicals with over 30,000 different isolated, synthesized, and identified compounds [46]. They comprise six C5 isoprene units and are biosynthesized by the cyclization of squalene [47,48,49]. Triterpenes are divided into groups based on the number of cyclic structures they contain [46]. The characteristic triterpene groups in *V. cinerea* are oleanane- and ursane-type triterpenes. They and their derivatives have exhibited various important biological and pharmacological activities, including anti-inflammatory, antiviral, cytotoxic, antimicrobial, and cardiovascular effects [50,51,52]. Compound 24-hydroxytaraxer-14-ene (**64**), the first identified oleanane-type triterpene of *V. cinerea*, was purified from the benzene extract of the air-dried and powdered roots of a specimen collected in Gorakhpur, India by Misra et al. in 1984 [53]. Similarly, six triterpenes (**57**–**62**) were also isolated from the roots of *V. cinerea* collected in India. Among these, 3*β*-acetoxyurs-13(18)-ene (**60**) had been synthesized before, but this was the first time it had been isolated from natural sources and analyzed by spectrophotometry [54]. In 1993, Misra et al. [55] added to their published research on the root compounds of *V. cinerea*. By nuclear magnetic resonance (NMR) spectroscopy and chemical degradation analyses, a new ursane-type triterpene named 3*β*-acetoxyurs-19-ene (**55**), together with a known triterpene, lupeol acetate (**56**), were isolated and structurally characterized from an ethanol extract of *V. cinerea* roots.

### 5.3. Steroids

Plants may contain a small amount of cholesterol, but their most common sterols are phytosterols, which are variants of cholesterol. Cholesterol and phytosterols both have a tetracyclic ring structure with a side chain at C-17, but the side chain in phytosterols is alkylated at C-24 with a methyl or ethyl substituent, and some of them, such as stigmasterol, contain double bonds [56,57]. In 1984, Misra et al. [58] reported the isolation of a new natural phytosterol named stigmast-5,17(20)-dien-3*β*-ol (**65**), along with stigmasterol (**66**) and sitosterol (**67**) from the roots of *V. cinerea* collected in India. In the same year, this group also described the isolation of two more phytosterols from the *V. cinerea* roots: campesterol (**68**) and *α*-spinasterol (**69**) [53]. The structures of these compounds are shown in Figure 4.

### 5.4. Flavonoids

Seven flavonoids (**70**–**76**) are known to be present in *V. cinerea* (Figure 5). Among them, most belong to the flavone and flavonol groups. Flavones are characterized by a C6-C3-C6 structure and possess two benzene rings, an oxygen-containing ring, and a C-2–C-3 double bond. Flavonols are similar, with the only difference being that flavonols have an additional hydroxy group in the C-3 position [59,60]. In 2013, Yadava et al. [61] reported two known compounds, luteolin (**70**) and taxifolin (**75**), and a new flavone glycoside from the roots of *V. cinerea* plants collected in the Sagar region of India. Employing various chemical degradations and NMR spectroscopy analysis, the new compound was identified as 5,7,4′-trihydroxy-3′-methoxyflavone-4′*-O-α*-L-rhamnopyranosyl-(1→4)*-O-α*-L-arabinopyranosyl-(1→3)*-O-β*-D-galactopyranoside (**76**), which exhibited moderate antiviral effects. In addition, several other flavonoids and flavonoid glycosides, including luteolin 4′-*O*-glucoside (**71**), luteolin 7-*O*-glucoside (**72**), quercetin 3*-O-β*-D-glucopyranoside (**73**), and apigenin (**74**), have been found in the roots of *V. cinerea* [27,45,62,63].

### 5.5. Phenolics and Other Compounds

Quinic acid derivatives and phenolic compounds were analyzed and reported for the first time in *V. cinerea* by Abeysekera et al. in 1998 [27]. Four quinic acid derivatives—chlorogenic acid (**77**), 3,5-dicaffeoylquinic acid (**78**), 3,4-dicaffeoylquinic acid (**79**), and 4,5-dicaffeoylquinic acid (**80**)—and a phenolic compound, methyl caffeate (**81**), were isolated from the aerial parts of the plant. In 2016, Youn et al. [63] also isolated and structurally characterized three phenolic compounds—(*E*)-4-(3,4-dimethoxyphenyl)but-3-en-1-ol (**82**) and 3-hydroxy-1-(4-hydroxy-3-methoxyphenyl)-propan-1-one (**83**)—and *trans*-cinnamic acid (**84**), together with 1*H*-indole-3-carbaldehyde (**87**) and uracil (**88**) from the aerial parts of *V. cinerea*. In addition, a new aliphatic acid, 26-methylheptacosanoic acid (**85**), was found in the roots of this species by Misra et al. in 1984 [58]. Recently, two new *trans-β*-bergamotene derivatives named (*E*)-*trans*-*β*-bergamotenol (**91**) and *trans-β*-bergamotenone (**92**) were isolated from the root and flower essential oils of *V. cinerea* [64]. This was the first report of novel bergamotene group compounds from this plant. The structures of these secondary metabolites are shown in Figure 6.

### 5.6. HPLC and GC-MS Identifications

Advanced techniques, such as HPLC (high-performance liquid chromatography) or GC-MS (gas chromatography–mass spectrometry), have been used in combination with traditional chromatography methods to determine the quantities and qualities of the chemical constituents in plants [65,66,67]. After demonstrating the antioxidant activities of a methanol extract of *V. cinerea* leaves, its phytochemical composition was analyzed using GC-MS, thus identifying 27 constituents. Gallic acid (1.92 mg/g) was the main phenolic component in the extract, as quantified by high-performance thin-layer chromatography (HPTLC), followed by rutin (0.705 mg/g), quercetin (0.173 mg/g), caffeic acid (0.082 mg/g), and ferulic acid (0.033 mg/g) [68]. In the same manner, 26 chemical components were identified in an essential oil of *V. cinerea* leaf and extracted using microwave-assisted hydrodistillation and Soxhlet extraction methods. These compounds included 9,12,15-octadecatrienoic acid (*Z,Z,Z*) (27.55%), 13-docosenoic acid methyl ester (20.02%), and *n*-hexadecanoic acid (8.55%) [69].

In an essential oil obtained from the roots of *V. cinerea*, 25 constituents were analyzed and identified by gas chromatography with flame ionization detection (GC-FID) and GC-MS, thereby representing 97.4% of the total oil. Among them, *α*-muurolene (30.7%) was quantified as the main compound, followed by *β*-caryophyllene (9.6%), *α*-selinene (8.7%), cyperene (6.7%) and *α*-gurjunene (6.5%) [70]. In addition, using GC-MS analysis, *trans-β*-bergamotene (20.7%), *β*-elemene (19.0%), and cyperene (10.6%) were identified and quantified from a root oil of *V. cinerea*, and *γ*-humulene (31.0%), (*E*)-*β*-caryophyllene (17.0%), and *trans*-*β*-bergamotene (7.7%) were identified and quantified from a flower oil [64].

**Table 1:** Secondary metabolites found in *V. cinerea*.

No.	Compound Name	Plant Part	References
*Sesquiterpene lactones*			
**1**	8*α*-Hydroxyhirsutinolide	Flowers, leaves, stems	[15,45]
**2**	8*α*-Hydroxyl-1-*O*-methylhirsutinolide	Flowers	[15]
**3**	8*α*-Tigloyloxyhirsutinolide	Flowers, leaves, stems	[15,16,44,45,71,72]
**4**	Vernolide-A	Flowers, leaves, stems	[15,42,44,45,71,72]
**5**	8*α*-(2′*Z*-tigloyloxy)-hirsutinolide	Leaves, stems	[45]
**6**	8*α*-(2′*Z*-tigloyloxy)-1*α*-methoxyhirsutinolide	Aerial parts	[73]
**7**	8*α*-(4-Hydroxytigloyloxy)-hirsutinolide	Leaves, stems	[45,72]
**8**	8*α*-(2-Methylacryloyloxy)-hirsutinolide	Aerial parts, leaves	[44,45,71,72]
**9**	8*α*-(2-Methylacryloyloxy)-1*α*-methoxyhirsutinolide	Aerial parts, leaves	[44,71]
**10**	Cyanolide D	Aerial parts	[44]
**11**	8*α*-Erucyl-1*α*-hydroxyl-hirustinolide	Leaves	[74]
**12**	8*α*-Hydroxy-13-*O*-tigloyl-hirsutinolide	Leaves, stems	[45]
**13**	Hirsutinolide-13-*O*-acetate	Leaves, flowers	[15,45,72,74]
**14**	Vernolide E	Whole plant	[72]
**15**	Piptocarphin D	Aerial parts	[16]
**16**	8*α*-Tigloyloxyhirsutinolide-13-*O*-acetate	Flowers, leaves, stems	[15,16,40,43,45,71,72]
**17**	Vernolide-B	Flowers, leaves, stems	[15,42,43,45,71,72]
**18**	Cyanolide B	Aerial parts	[44]
**19**	Cyanolide C	Aerial parts	[44]
**20**	Piptocarphin B	Aerial parts	[44]
**21**	8*α*-(2′*Z*-tigloyloxy)-hirsutinolide-13-*O*-acetate	Leaves, stems	[45]
**22**	Vernolide F	Whole plant	[72]
**23**	8*α*-(4-Hydroxytigloyloxy)-hirsutinolide-13-*O*-acetate	Aerial parts	[16,40,71,72]
**24**	Vernolide G	Whole plant	[72]
**25**	8*α*-(4-Hydroxytigloxyloxy)-10*α*-hydroxyhirsutinolide-13-*O*-acetate	Aerial parts	[40,71]
**26**	8*α*-(2-Methylacryloyloxy)-hirsutinolide-13-*O*-acetate	Flowers, leaves, stems	[15,43,45,71,72]
**27**	8*α*-(2-Methylacryloyloxy)-1*α*-methoxyhirsutinolide-13-*O*-acetate	Flowers	[15,43,72]
**28**	8*α*-(4-Hydroxymethacryloyloxy)-hirsutinolide-13-*O*-acetate	Aerial parts	[16,40,72]
**29**	8*α*-(4-Hydroxymethacryloyloxy)-10*α*-hydroxyhirsutinolide-13-*O*-acetate	Aerial parts	[40]
**30**	8*α*-(2′-Hydroxymethylacryloyloxy)-1*α*-methoxyhirsutinolide-13-*O*-acetate	Aerial parts	[72,73]
**31**	Vernolide I	Whole plant	[72]
**32**	Vernolide J	Whole plant	[72]
**33**	Vernolide C	Aerial parts, leaves	[16,44]
**34**	8*α*-Epoxymethacryloyloxy-hirsutinolide-13-*O*-acetate	Aerial parts	[16]
**35**	(1S*,4R*,8S*,10R*)-1,4-epoxy-13-ethoxy-1,8,10-trihydroxygermacra-5*E*,7(11)-dien-6,12-olide	Aerial parts	[44]
**36**	Vernobockolide B	Whole plant, leaves	[44,72]
**37**	Cyanolide A	Aerial parts	[44]
**38**	Stilpnotomentolide-8-*O*-tiglate	Aerial parts	[40]
**39**	Vernocinerolide-8-*O*-(4-hydroxymethacrylate)	Aerial parts	[40]
**40**	Glaucolide E	Aerial parts	[40]
**41**	19-Hydroxyglaucolide E	Aerial parts	[40]
**42**	Vernocinolide A	Aerial parts	[71]
**43**	8*β*-[benzoic acid]-4*β*,6*α*,10*β*,13-tetrahydroxyl-7(11)-guaiaen-12,6-olide	Leaves	[74]
**44**	Vercinolide A	Whole plant	[43]
**45**	Vercinolide B	Whole plant	[43]
**46**	Vercinolide C	Whole plant	[43]
**47**	Vercinolide D	Whole plant	[43]
**48**	Vercinolide E	Whole plant	[43]
**49**	Vercinolide F	Whole plant	[43]
**50**	Vercinolide G	Whole plant	[43]
**51**	Vercinolide H	Whole plant	[43]
*Norisoprenoids*			
**52**	Loliolide	Leaves, stems	[45]
**53**	Isololiolide	Leaves, stems	[45]
**54**	(3*R*)-3-Hydroxyionone	Leaves, stems	[45]
*Triterpenes*			
**55**	3*β*-Acetoxyurs-19-ene	Roots	[55]
**56**	Lupeol acetate	Roots	[55]
**57**	*δ*-Amyrin acetate	Roots	[54]
**58**	*α*-Amyrin acetate	Roots	[54]
**59**	*β*-Amyrin acetate	Roots	[54,75]
**60**	3*β*-Acetoxyurs-13(18)-ene	Roots	[54]
**61**	*α*-Amyrin	Roots	[54]
**62**	*β*-Amyrin	Roots	[54]
**63**	Lupeol	Leaves	[75,76]
**64**	24-Hydroxytaraxer-14-ene	Roots	[53]
*Steroids*			
**65**	Stigmast-5,17(20)-dien-3*β*-ol	Roots	[58]
**66**	Stigmasterol	Roots	[58,75]
**67**	Sitosterol	Roots	[58,75]
**68**	Campesterol	Roots	[53]
**69**	*α*-Spinasterol	Roots	[53]
*Flavonoids*			
**70**	Luteolin	Aerial parts, roots	[61,62]
**71**	Luteolin 4′-*O*-glucoside	Aerial parts	[27]
**72**	Luteolin 7-*O*-glucoside	Aerial parts	[62]
**73**	Quercetin 3-*O*-*β*-D-glucopyranoside	Aerial parts	[63]
**74**	Apigenin	Leaves, stems	[45]
**75**	Taxifolin	Roots	[61]
**76**	5,7,4′-Trihydroxy-3′-methoxyflavone-4′*-O-α*-L-rhamnopyranosyl-(1→4)*-O-α*-L-arabinopyranosyl-(1→3)*-O-β*-D-galactopyranoside	Roots	[61]
*Phenolic compounds*			
**77**	Chlorogenic acid	Aerial parts	[27]
**78**	3,5-Dicaffeoylquinic acid	Aerial parts	[27]
**79**	3,4-Dicaffeoylquinic acid	Aerial parts	[27]
**80**	4,5-Dicaffeoylquinic acid	Aerial parts	[27]
**81**	Methyl caffeate	Aerial parts	[27]
**82**	(*E*)-4-(3,4-Dimethoxyphenyl)but-3-en-1-ol	Aerial parts	[63]
**83**	3-Hydroxy-1-(4-hydroxy-3-methoxyphenyl)-propan-1-one	Aerial parts	[63]
**84**	*trans*-Cinnamic acid	Aerial parts	[63]
*Other compounds*			
**85**	26-Methylheptacosanoic acid	Roots	[58]
**86**	(9*Z*,12*S*,13*S*)-Dihydroxy-9-octadecanoic acid	Leaves, stems	[45]
**87**	1*H*-Indole-3-carbaldehyde	Aerial parts	[63]
**88**	Uracil	Aerial parts	[63]
**89**	(*E*)-*trans*-*α*-Bergamotenol	Flowers, roots	[64]
**90**	*trans-β*-Bergamotene	Flowers, roots	[64]
**91**	(*E*)-*trans-β*-Bergamotenol	Flowers, roots	[64]
**92**	*trans–β*–Bergamotenone	Flowers, roots	[64]

## 6. Pharmacological Activities

**Figure 7:**
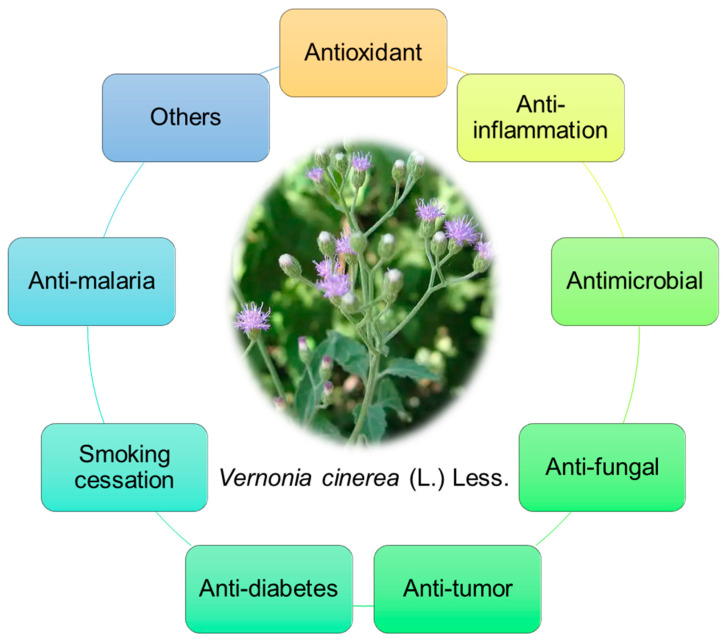
Pharmacological activities of *V. cinerea*.

Recently, the various pharmacological properties of the extracts and isolated compounds of *V. cinerea* have been studied in vitro and in vivo. Among the studied effects, the traditional and ethnopharmacological uses of this plant for its anti-inflammatory, antiprotozoal, antidiabetic, anticancer, antimicrobial, antioxidant, and renoprotective activities have found scientific support. A summary of the pharmacological activities is shown in Figure 7.

### 6.1. Antioxidant Activity

Several studies have reported that *V. cinerea* shows promising antioxidant activity. Using several protocols, such as 2,2-diphenyl-1-picrylhydrazyl (DPPH) and 2,2′-azino-bis (3-ethylbenzothiazoline-6-sulfonic acid) (ABTS) radical scavenging activity assays, an ethanol extract of *V. cinerea* was shown to have an activity of 16.48 mg gallic/g extract [77]. Additionally, other studies indicate that the methanol extract of leaves and flowers exhibits approximately 70% DPPH inhibition [78], and the concentration of stembark and leaf extract needed for 50% scavenging (IC_50_) of DPPH was found to be 82 ± 3.40 µg/mL [79]. Also, there are many compounds isolated from *V. cinerea* that have potential antioxidant activities. For example, lupeol, gallic acid, and quercetin produced IC_50_ values against DPPH activity of 30, 0.62, and 0.53 µg/mL, respectively [76,80]. Also, the leaf extract of *V. cinerea* exhibited antioxidant activity at 117.71 ± 15.02 µM Trolox equivalents/100 mg of dry extract [81].

A recent study indicated an alternative modern technique for the nitrate extraction of *V. cinerea* whole plants using microwave-assisted extraction. Using a microwave power of 300 W, a duration of 10 s, and one irradiation cycle, a whole plant extraction yield of 15.9 ± 0.2% and nitrate content of 1.32 ± 0.01% was achieved. Using this extract, the IC_50_ of a DPPH assay was 0.4 mg/mL, while the IC_50_ of a ferric-reducing antioxidant power assay was nearly 0.4 mg/mL [82].

### 6.2. Anti-Inflammatory Activity

Previous studies have investigated nine compounds isolated from the flowers of *V. cinerea* for their anti-inflammatory activity. Among them, vernolide-A (**4**), hirsutinolide-13-*O*-acetate (**13**), 8*α*-tigloyloxyhirsutinolide-13-*O*-acetate (**16**), vernolide-B (**17**), and 8*α*-(2-methylacryloyloxy)-1*α*-methoxyhirsutinolide-13-*O*-acetate (**27**) suppressed nitric oxide (NO) production and tumor necrosis factor alpha (TNF-*α*)-induced NF-κB activity [15,43,72]. Other research has focused on the anti-inflammatory effect of *V. cinerea* extracts in vivo using the acute inflammatory model of carrageenin-induced paw edema in Wistar albino rats. Their findings indicate that the chloroform, methanolic, and petroleum ether extracts of leaves have potent and significant suppressant activities [9,83]. Another study showed that *V. cinerea* extract showed remarkably suppression against carrageenan injection paw edema in BALB/c mice and downregulated the expression of proinflammatory cytokines, including level TNF-*α*, interleukin (IL)-1*β*, and IL-6, and in lipopolysaccharide (LPS)-activated macrophages [84]. Additionally, the methanolic extract of *V. cinerea* induced an enhancement in the phagocytic activity of peritoneal macrophages, downregulated inducible NO synthase and cyclooxygenase-2 (COX-2) mRNA levels in LPS-activated macrophages, and inhibited the proinflammatory cytokines interferon gamma (IFN-γ) and IL-2, while it selectively increased T-helper 2 (Th2) cell-related cytokine secretion, thus demonstrating that the extract causes Th2 polarization [85,86]. Also, an *n*-hexane extract of *V. cinerea* stems, at 12.5 µg/mL, significantly reduced IL-6 levels by 57.89 ± 2.54%, and an immunocytochemistry analysis suggested that this may have been due to the inactivation of NF-κB signaling through the inhibition of nuclear translocation [87]. In addition, in smokers receiving three lozenges per day, an extract of the aerial parts of *V. cinerea* combined with a leaf extract of *Moringa oleifera* Lam. produced an antioral inflammatory effect, thus resulting in a 21.28% drop in the gingival index and a 57.14% decrease in oral inflammation [81].

### 6.3. Antipyretic Activity

Methanol, chloroform, and ether extracts of *V. cinerea* leaves have been reported to have antipyretic effects in vivo at doses of 100, 200, and 400 mg/kg, intraperitoneally (IP). Rats injected with brewer’s yeast exhibited a significant increase in rectal temperature within 18 h. No significant antipyretic effect was seen after administering a petroleum ether extract of *V. cinerea*, while an methanolic extract at the dose of 100 mg/kg showed a significant impact 30 min after administration and onward, reducing temperatures from 38.0 ± 0.1 °C to 37.0 ± 0.3 °C, and chloroform extract at 400 mg/kg significantly reduced pyrexia starting at 2 h after administration, dropping the mean temperature from 37.4 ± 0.1 °C to 36.6 ± 0.4 °C. Treatment with paracetamol (100 mg/kg orally) started reducing the temperature only at 1 h after administration and only from 37.4 ± 0.1 °C to 37.2 ± 0.2 °C [83]. Another study indicated that a whole-plant methanol extract of *V. cinerea* displays antipyretic potential against yeast-induced pyrexia in rats at doses of 250 and 500 mg/kg of body weight (BW), taken orally, after 2 h [88].

### 6.4. Cholinesterase Inhibition

There are two types of cholinesterases, acetylcholinesterase (AChE) and butyrylcholinesterase (BChE), that are attractive as candidate targets for the treatment of Alzheimer’s disease [89,90]. The methanolic leaf extract of *V. cinerea* significantly inhibits the effects of AChe and BChE, with IC_50_ values of 160.5 ± 1.1 μg/mL and 205.4 ± 2.2 μg/mL, respectively, compared to the positive control, eserine, which has an IC_50_ of 0.018 ± 0.01 μg/mL for AChE inhibition and 0.038 ± 0.01 μg/mL for BChE inhibition [79].

### 6.5. Antitumor Activities

Radiation plays a vital role in the treatment of cancer, but radiation therapy is fraught with serious side effects, one of which is normal tissue damage [91]. An in vivo study reported that a methanolic extract of *V. cinerea* produced significant radioprotective activity against gamma radiation-induced immunosuppression in irradiated BALB/c mice. Particularly, the total white blood cell count was efficiently maintained at an extract dose of 20 mg/kg BW IP. On day eleven of the radiation therapy, it was discovered that the scavenging activity of the *V. cinerea* extract had returned lipid peroxidation to normal levels in the treated animals. Additionally, the *V. cinerea* extract markedly increased the endogenous glutathione levels in the gut and liver, and the hepatoprotective potential of *V. cinerea* was demonstrated by its ability to reduce high radiation-induced levels of alkaline phosphatase and glutamate pyruvate transferases in both the liver and serum. Ultimately, the extract treatment decreased liver damage and the amount of DNA damage in the bone marrow cells of the irradiated mice [8].

The kidney damage caused by cisplatin, a chemotherapy drug [92], was significantly reversed in vivo by a crude aqueous extract of *V. cinerea*. Ehrlich ascites carcinoma (EAC)-bearing mice treated with a butanol fraction and crude aqueous extract demonstrated a 50–75% regeneration of proximal tubular cells. The life span of the cisplatin-treated group was 244% that of the EAC control mice, and when the crude aqueous extract was administered, the life span further increased to 379%. Thus, compared to the group treated with cisplatin alone, the group treated with the extract showed a 1.6-fold increase in life span [93]. Previous studies have revealed that free radicals are a crucial factor in the development of nephrotoxicity [94,95]. Sreedevi et al. [96] indicated that petroleum ether, ethyl acetate, and alcoholic extracts from the aerial parts of *V. cinerea* (500 mg/kg, taken orally) have a protective effect on cisplatin (6 mg/kg IP)-induced nephrotoxicity in albino rats without any deteriorative effects on the kidney. Pretreatment with *V. cinerea* extract decreased blood urea nitrogen, serum creatinine, serum total proteins, and urinary proteins in vivo. In another study, *V. cinerea* was shown to shield BALB/c mice against the toxicity caused by cyclophosphamide. Comparing *V. cinerea*-treated animals with control mice, the intraperitoneal administration of an extract resulted in a significant rise in the total white blood cell count, bone marrow cellularity, esterase-positive cells, and the weights of lymphoid organs [97].

*V. cinerea* also exhibits antiproliferative activities. A study reported that *V. cinerea* inhibits the proliferation of HT-1080 human fibrosarcoma tumor cell line cells [98]. *V. cinerea* extract also significantly inhibited lung tumor formation (by 78.8%) in B16F-10 melanoma cells by suppressing the production and expression of proinflammatory cytokines such as TNF-*α*, IL-1*β*, IL-6, and granulocyte-macrophage colony-stimulating factor (GM-CSF) [99]. Additionally, it suppresses the expression of matrix metalloproteinase (MMP)-2, MMP-9, lysyl oxidase, prolyl hydroxylase, K-ras, extracellular signal-regulated kinase (ERK)-1, ERK-2, and VEGF while increasing the expression of Non-metastatic protein 23 (Nm-23), tissue inhibitor of metalloproteinase (TIMP)-1 and TIMP-2 in vivo [99]. The ethyl acetate extract of *V. cinerea* reportedly exhibited toxicity against Dalton’s lymphoma ascites (DLA) cells, with a lethal concentration required to kill 50% of the population exposed (LC_50_) of 61.24 μg/mL. Furthermore, the antiproliferative effect of this extract on tumor cells was demonstrated through the long-term incubation of YAC-1 cells, another T-cell lymphoma cell line, with varying doses of the extract. A GC-MS analysis revealed the presence of nerolidol, a volatile molecule, with the LC_50_ value increasing from 2.27 µM to 20.50 µM when treated with deferoxamine. Additionally, in vivo studies using the DLA-induced solid tumor model showed that an ethyl acetate extract of *V. cinerea* led to a 69.9% reduction in tumor size compared to the positive control: cyclophosphamide (10 mg/kg BW) [10].

In a study assessing the effect of *V. cinerea* on mice infected with several cancer types, it was found that, after receiving 2000 mg/kg BW of crude extract, the animals did not exhibit any toxic symptoms or death, thus living for up to 14 days. At the final stage of the experiment, an autopsy on the heart, kidneys, liver, lungs, and spleen showed no signs of abnormalities. Nevertheless, human κB (oral epidermoid carcinoma), DLD-1 (colon adenocarcinoma), NCI-661 (lung large cell carcinoma), and Hela (cervix epithelioid carcinoma) tumor cell lines were all resistant to two active sesquiterpene lactones: vernolide-A and -B (**4** and **17**). Notably, compound **4** exhibited potent cytotoxicity against the human tumor cell lines DLD-1, Hela, NCI-661, and κB (ED_50_ = 0.02, 0.05, 0.53, and 0.04 mg/mL, respectively), while compound **17** showed only moderate cytotoxicity against these cancer cell lines (ED_50_ = 3.78, 5.88, and 6.42 mg/mL for κB, NCI-661, and Hela, respectively) [42,100]. Using a C57BL/6 mouse model, the effect of compound **4** on the cell-mediated immune response under metastatic conditions was investigated. Increased natural killer cell activity was observed in both tumor-bearing and normal animals. The IL-2 and IFN-γ production in mice with metastatic tumors was markedly increased by the administration of compound **4**. Furthermore, during the metastasis of B16F-10 melanoma cells in mice, compound **4** dramatically reduced the serum levels of proinflammatory cytokines such IL-1*β*, IL-6, TNF-*α*, and GM-CSF [101,102].

The sesquiterpene lactone 8*α*-tigloyloxyhirsutinolide-13-*O*-acetate (**16**), a secondary metabolite isolated from various parts of *V. cinerea*, significantly reduced the growth of cancer cells in cell line HSC4 compared to cell line A549 and markedly lowered the proliferation of cancer cells in normal oral cells. In both cancerous cell lines, compound **16** suppressed the phosphorylation of STAT3. Interestingly, this active compound could inhibit the persistently activated STAT2 state exclusive to HSC4. Cyclin B1 was found to be downregulated, resulting in G2/M cell cycle arrest, as a consequence of compound **16**′s suppression of STAT2. This, in turn, led to the downregulation of ISG15 and ISG15 conjugates, thereby reducing the expression of CDK1/2 [103]. Molecular dynamics simulations, a leading research tool for discovering and developing targeted protein inhibitors [104], indicated that a lung cancer-associated mutation in the gene of epidermal growth factor receptor (EGFR), resulting in a substitution of Leu 858 to Arg (L858R), can be effectively treated with medicines that bind to EFGR, including luteolin-7-glucoside (**72**) and epicatechin gallate from *V. cinerea* because of their superior inhibitory qualities [105]. The stability of epicatechin gallate and **72** with EGFR-L858R were analyzed in explicit water conditions employing a 60 ns molecular dynamics trajectory. When comparing EGFR-L858R to PD168393, an EGFR inhibitor, the analysis of hydrogen bonding patterns, radius of gyration, conformational element deviations, residual component fluctuations, and solvent-accessible surface area revealed that both active compounds exhibited high stability in the binding site of the target protein [105]. In breast cancer or glioblastoma cells, compound **16** and 8*α*-(2′Z-tigloyloxy)-hirsutinolide-13-*O*-acetate (**21**) reportedly inhibited aberrant STAT3 activity. In addition, compounds **16**, **17**, and **21** affected the vitality of the U251MG glioblastoma cell line [45]. Normal human epithelial cells were less affected, but human adenocarcinoma cells showed dose-dependent cytotoxicity in response to the dichloromethane fraction of *V. cinerea*. When combined with anticancer medications, “sesquiterpenoids”-enriched fractions dramatically reduced the functional activity of MDR transporters (ABCB1 and ABCG2) and produced “synergistic cytotoxic effects” in human adenocarcinoma cells [106]. Additionally, hirsutinolide-type sesquiterpenoids from *V. cinerea* exhibited IC_50_ values against human prostate (PC-3) and LNCaP cancer cell lines ranging from 2.2 ± 0.4 to 8.5 ± 0.7 μM and 3.0 ± 0.7 to 10.5 ± 1.1 μM, respectively, thus indicating substantial antiprostate cancer activity [44]. Also, compound **11** showed anticancer activity with IC_50_ values of 12.5 and 10.4 μg/mL against human renal cell carcinoma (786-O cell line) cells after 48 and 72 h, respectively [74].

### 6.6. Hepatoprotective Activity

Since the liver is the principal organ involved in the metabolism of drugs and hazardous chemicals, it is the organ that almost all toxins target first [107]. Carbon tetrachloride (CCl_4_), a strongly hepatotoxic chemical capable of inducing both acute and chronic liver injury in animals, has been employed to induce hepatic necrosis. Animals exposed to CCl_4_ display elevated levels of liver marker enzymes in their serum, thus indicating hepatic cell damage [108]. Acute toxicity induced by CCl_4_ results in enhanced cellular leakage and increased permeability of hepatocyte membranes. However, the elevated activities of liver marker enzymes were suppressed by *V. cinerea* extract, thus suggesting that the extract might offer protection against liver damage following CCl_4_ induction [109].

### 6.7. Diuretic Effect and Antiurolithiasis Activity

A previous study revealed that *V. cinerea* can cure urinary incontinence. A chloroform extract of *V. cinerea* leaves exhibited effective hypernatremia, hyperkalemic, and hyperchloremic diuretic effects. Meanwhile, methanol and aqueous extracts of *V. cinerea* showed antidiuretic effect in vivo. At more than 62.5 mg/kg dosages, the extracts caused a dose-dependent increase or decrease in urine volume with a significant dose-dependent increase or decrease in the excretion of Na^+^ and K^+^ and a significant, non-dose-dependent excretion of Cl^−^. In comparison, the common diuretic furosemide (10 mg/kg) and standard antidiuretic chlorpropamide (10 mg/kg) caused less excretion of electrolytes and less increases or decreases in urine volume [110].

The formation and retention of stones in several areas of the urinary tract, including the kidney, ureter, and bladder, with a considerable variation in calculus size and type, is known as urolithiasis [111]. It was reported that the hydroalcoholic extract of *V. cinerea* may have the potential to prevent and cure urolithiasis in vivo, which was demonstrated using an ethylene glycol-induced model at doses of 100, 200, and 400 mg/kg BW by oral administration [112].

### 6.8. Antidiarrheal Effects

The methanol extract of stembark and leaves of *V. cinerea* were reported to have anti-diarrheal activity in castor-induced diarrhea in Swiss albino mice [11]. However, the *V. cinerea* leaf extract at oral doses of 250 and 500 mg/kg BW did not possess antidiarrheal activity.

### 6.9. Antifeedant Effects

Compounds **66** and **72** isolated from *V. cinerea* have demonstrated significant antifeedant activity against two lepidopterous insects, *Spodoptera litura* and *Spilosoma obliqua*, based on percent feeding deterrence and effective doses (ED_50_). At a dosage of 3000 ppm, compound **72** showed 98.41% and 98.61% feeding deterrence against *S. obliqua* and *S. litura*, with ED_50_ values of 432.96 ppm and 586.95 ppm, respectively. Whereas, at a concentration of 5000 ppm, compound **66** demonstrated 94.84% and 94.38% feeding deterrence against these two insects, with ED_50_ values of 557.14 ppm and 964.10 ppm, respectively [113].

### 6.10. Antidiabetic Activity

The methanolic extract of *V. cinerea* stembark and leaves reportedly suppressed alloxan-induced diabetes in vivo at doses of 250 and 500 mg/kg, thus significantly reducing blood glucose levels in alloxan-induced diabetic rats compared to the control group [11]. Remarkably, by influencing the phosphatidylinositol-3-kinase (PI3K) and adenosine monophosphate-activated protein kinase (AMPK) pathways in the liver, skeletal muscle, and adipose tissue, *V. cinerea* water extract improved insulin sensitivity in obese mice generated by high-fat diets (HFDs). Mice were fed an HFD of 188.28 kJ (45 kcal% lard fat) for 12 weeks to produce obesity. Treatment with a *V. cinerea* water extract at 250 and 500 mg/kg BW doses was administered to obese mice during the final six weeks of the HFD. Hyperglycemia, hyperinsulinemia, hyperleptinemia, and hyperlipidemia were all markedly lowered by the extract treatment at both dosages. The treatment resulted in a rise in serum adiponectin but a drop in TNF-*α*, monocyte chemoattractant protein-1, and proinflammatory cytokines. The *V. cinerea* water extract also lowered the number of triglycerides stored in the skeletal muscle and liver and decreased the average fat cell size of the obese mice. By increasing the phosphorylation of PI3K, protein kinase B, AMPK, and acetyl-CoA carboxylase, *V. cinerea* water extraction therapy enhanced protein expressions in the PI3K and AMPK pathways in the liver, skeletal muscle, and adipose tissue. Additionally, in obese mice, the extract increased glucose transporter 4 in both muscle and adipose tissues [114].

Additionally, an ethanol extract from the leaves and stems of *V. cinerea* restored testicular function and testosterone concentration in male rats with streptozotocin (60 mg/kg, IP)-induced diabetes mellitus. In diabetes mellitus rats, an extract pretreatment at 10 and 40 mg/kg BW dramatically improved testosterone concentrations and sperm motility while reducing testicular histological changes. Additionally, their sperm counts significantly increased, and an antidiabetic effect was observed at an extract dosage of 40 mg/kg [115].

In a clinical trial, a randomized, single-center, double-blind, and crossover study was conducted on patients with type 2 diabetes mellitus using an herbal preparation containing *V. cinerea*. The results showed significant reductions in fasting blood glucose, hemoglobin A1C, cholesterol, LDL cholesterol, and triglyceride levels, thus supporting the amelioration of the glycemic state in patients with type 2 diabetes. This effect was observed with a dosage of 6 g/day (administered as one 2 g pill three times daily) over a 6-month therapy period [116].

### 6.11. Antiprotozoal and Larvicidal Activity

Methanolic and water extracts of *V. cinerea* have also been shown to have an antiprotozoal effect on *Leishmania donovani* promastigotes, thereby achieving IC_50_ values of 181.92 ± 1.15 and 443.61 ± 2.35 µg/mL, respectively [117].

The larvicidal efficacies of ethyl acetate, chloroform, acetone, and methanol leaf extracts of *V. cinerea* against the common filarial vector, *Culex quinquefasciatus*, were also investigated. The ethyl acetate was found to be the most effective, with an LC_50_ value of 1.63 mg/mL after 24 h, followed by the chloroform, acetone, and methanol extracts, with LC_50_ values of 1.84, 1.89, and 2.08 mg/mL, respectively [118]. Also, against *Aedes albopictus*, a vector of dengue and chikungunya, an acetone extract of *V. cinerea* leaf powder exhibited a significant efficacy, thus producing an LC_50_ value of 0.22 g/L and LC_90_ value of 0.96 g/L [119].

### 6.12. Antimalarial Activity

Chea et al. [16] reported that three sesquiterpene lactones **3**, **16**, and **23** were active against a chloroquine-resistant *Plasmodium falciparum* strain, thereby showing IC_50_ values of 3.7, 3.9, and 3.5 mM, respectively. Notably, lactone-enriched extract, created through fraction enrichment by the liquid–liquid portioning of *V. cinerea* extract and synthesis of mediated nanogold composites (LEF-AuNPs) in a single-step process, revealed potential antimalarial activity. Higher doses of LEF-AuNPs reduced parasitemia significantly by the eighth day after infection compared to untreated mice, with the highest reduction at 29.09%. Mice treated with LEF-AuNPs survived longer than those infected alone, especially those given 100 mg/kg, which survived 10 days [120].

Additionally, the ethnology of *V. cinerea* has an impact on the antimalarial activity of the plant. Dichloromethane and methanol whole-plant extracts of *V. cinerea* collected from Cambodia were reported to have antiplasmodial activity against *Plasmodium falciparum* type W2 strain, with IC_50_ values of 18.3 and 32.1 g/mL, respectively [121]. Furthermore, root and whole-plant extracts collected from India exhibited 55 and 65% inhibition at the concentration of 100 ug/mL [122].

### 6.13. Antifungal and Antimicrobial Activity

Many fungi have been identified as plant pathogens. Fungal diseases frequently reduce crop yields and quality by producing toxins that are hazardous to human health. Fungal infection is a very serious cosmetic problem that is of utmost concern globally, and one such infection is dandruff [123]. The ethanol extract of *V. cinerea* leaves exhibited broad antifungal activity on the growth of *Cercosporell apersica* and *Curvularia lunatus* in vitro. After 7 days, *V. cinerea* extract produced 80% inhibition at a concentration of 200 mg/mL and reached 100% inhibition against *C. persica* at 300 mg. It also inhibited 69.77% of the growth of *C. lunatus* at an extraction dosage of 200 mg and reached 100% inhibition of *C. lunatus* growth at a concentration of 300 mg after 7 days [124]. In addition, with a minimum inhibitory concentration (MIC) of 1.56 mg/mL, the methanolic leaf extract of *V. cinerea* exhibited a strong antimicrobial activity against *Candida albicans* [125]. Using a microscope to observe the extract for 36 h, the time-kill experiment revealed that *V. cinerea* extract had inhibited *C. albicans* growth and had persistent antiyeast action. It also showed favorable antimicrobial activity against *Pseudomonas aeruginosa*, with an MIC value of 3.13 mg/mL [126]. The methanol extract of *V. cinerea* reportedly inhibited the growth of *Xanthomonas oryzae* pv. *oryzae* bacteria after 24 h of incubation, thus creating a notable zone of inhibition [127]. At a concentration of 25 μg/mL, the zone measured 16.0 mm. Increasing the concentration to 50 μg/mL yielded a zone size of 18.1 mm, while at 100 μg/mL, the zone size was 22.6 mm. In contrast, the positive control, tetracycline, at a concentration of 5 μg/mL, produced a zone of inhibition measuring 33.17 mm. The MIC for *V. cinerea* methanol extract against *X. oryzae* was determined to be 10 μg/mL compared to 1 μg/mL for tetracycline.

Additionally, it was discovered that the ethyl acetate extract of *V. cinerea* had potential antifungal activity against *Candida parapsilosis*, with zones of inhibition measuring 12, 13, 16, and 20 mm at concentrations of 250, 500, 750, and 1000 μg/mL, respectively. It also demonstrated remarkable antidandruff properties against *Pityrosporum folliculitis* and *P. ovale*, with zones of inhibition measuring 19 and 20 mm, respectively [128]. The benzene fraction of *V. cinerea* exhibited a broad spectrum of antibacterial activity against *Bacillus subtilis*, *Staphylococcus aureus*, *S. epidermidi*, *Micrococcus luteus*, *Escherichia coli*, *Klebsiella pneumoniae*, *Salmonella typhi*, *Shigella dysenteriae*, and *Psudomonas aeruginosa*, with zones of inhibition ranging from 9 to 18 mm at a concentration of 250 μg/mL, and from 21 to 28 mm at a concentration of 500 μg/mL [6]. Furthermore, an aqueous extract of *V. cinerea* showed MIC values of 49.6 and 49.6 mg/mL against *Streptococcus mutans* serotypes c (MT50911) and d (OMZ1761) [129], and a 500 mg/mL aqueous extract showed produced zones of inhibition against *S. aureus* and methicillin-resistant *S. aureus* of 13.0 mm and 15.0 mm, respectively [77]. According to Sonibare et al. [80], the minimum bactericidal concentration (MBC) values for the *n*-hexane fraction of *V. cinerea* were 3.13, 12.5, 6.25, 6.25, 6.25, and 3.13 mg/mL against *S. aureus*, *E. coli*, *K. pneumoniae*, *P. aeruginosa*, *Proteus vulgaris*, and *C. albicans*. The chloroform fraction had MBC values of 6.25, 12.5, 6.25, 6.25, 3.13, and 3.13 mg/mL against the same strains. The ethyl acetate fraction had MBC values of 12.5, 6.25, 3.13, 12.5, 3.13, and 3.13 mg/mL.

Other studies reported that, compared to Gram-positive *B. subtilis*, seeds of *V. cinerea* have more potency against Gram-negative bacteria, including *E. coli*, *Pseudomonas cichorii*, and *Salmonella typhimurium* and have a potential effective against five *Helicobacter pylori* bacterial strains, including BCRC 17026, BCRC 15415, Qu 108, Qu 141, and Qu 150, with zones of inhibition ranging from 11 to 20 mm at a concentration of 0.2 g/mL [130,131].

### 6.14. Antiviral Activity

A flavone glycoside **76** isolated from the roots of *V. cinerea* reportedly exerted antiviral effect on the Japanese encephalitis virus in vitro, thus achieving an antiviral activity of 50% [61].

### 6.15. Antiarthritic Activity

The extract of *V. cinerea* flower suppresses adjuvant arthritis induced by the intradermal injection of complete Freund’s adjuvant in female albino Wistar rats [132]. During 19 days of the experimental period, the body weight of rats recovered in the orally administered *V. cinerea* group compared to the arthritis group. The paw volume decreased, thus showing consistency with the serum and tissue aminotransferases in liver, kidney, spleen, and serum.

### 6.16. Analgesic Effect

Chloroform, methanolic, and petroleum ether leaf extracts of *V. cinerea* were shown to have effective analgesic activities at 100, 200, and 400 mg/kg IP, which dramatically enhanced mechanically caused pain by analgesia meter and significantly decreased pain induced by acetic acid writhing responses on the edematous rat paw [83]. The number of writhing episodes in treated mice considerably reduced in the chloroform extract-, methanolic extract-, and petroleum ether extract-treated groups at the dose of 400 mg/kg BW was 36.0 ± 1.0, 26.4 ± 6.1 and 12.8 ± 2.4, respectively, compared to the saline-treated group, which was 55.3 ± 1.7 every 20 min. In mechanical-induced pain, there was an increase in pain threshold, as exhibited by the three extracts when compared with the control group. Also, the three extracts demonstrated higher pain thresholds in mechanically produced pain than in the control group.

### 6.17. Effective in Smoking Cessation

One of the leading causes of disease and early mortality in the world is smoking [133]. One-hundred and twenty people participated in the investigation of *V. cinerea* in relation to oxidative stress status and beta-endorphin release in active smokers by orally exhibiting positive results [134]. Total antioxidant capacity increased in the *V. cinerea* supplement group, whereas malondialdehyde, protein hydroperoxide, and nitric oxide significantly decreased. Following the intervention, CO levels were lower in all groups. The exercise-only group (53.57%), exercise with *V. cinerea* supplement group (40%), exercise with *V. cinerea* supplement group (62.7%), and control group (14.04%) all saw a decrease in the smoking rate for light cigarettes. In contrast, the smoking rate for self-rolled cigarettes decreased in the exercise-only group (42.30%), exercise with *V. cinerea* supplement group (40%), and control group (9.2%).

In clinical trials, five trials involving 347 smokers were included. The *V. cinerea* treatment group had significantly higher quit rates compared to the control group. At week 8, the continuous abstinence rate was 1.69 times higher (95% CI [1.00, 2.86]), and at week 12, it was 2.18 times higher (95% CI [1.17, 4.04]). Similarly, the 7-day point prevalence abstinence rate at week 8 was 1.51 times higher (95% CI [1.01, 2.27]), and at week 12, it was 1.93 times higher (95% CI [1.24, 2.99]) [135]. In addition, a community pharmacy conducted a double-blind controlled trial with 121 eligible volunteers. There were 111 eligible subjects in all; 54 received *V. cinerea* treatment (48.65%), while 57 received a placebo (51.35%). By the end of the trial, the *V. cinerea* group had a considerably higher chance of quitting smoking than the placebo group, with a difference of 2.01 (95% CI of 1.03, 3.92), and neither group experienced any severe side effects [12].

The main enzyme that breaks down nicotine ingested into the body is human liver cytochrome P450 2A6 (CYP2A6), a heme-containing enzyme in the cytochrome P450 monooxygenase superfamily [136]. Furthermore, CYP2A6 increases the risk of lung cancer and respiratory disorders in smokers by mediating the activation of tobacco-specific carcinogenic chemicals [137]. Flavonoids from *V. cinerea* were found to exhibit a significant degree of reversible inhibition on CYP2A6 [138,139]. When comparing the IC_50_ values of flavonoids from *V. cinerea* against human mitochondrial monoamine oxidases (MAO-A and MAO-B), responsible for dopamine metabolism, it was found that luteolin (**70**) significantly inhibited both MAO-A and MAO-B, with IC_50_ values of 4.01 and 0.97 mM, respectively [140].

### 6.18. Antianxiety Effect

When overused, sunset yellow, widely used in food and drink, can cause various negative effects on major organs, including the brain, liver, and urinary system [141]. According to a recent study, the extracts from the aerial parts of *V. cinerea* (400 mg/kg, taken orally) significantly enhances the effects of the neurotransmitter gamma-aminobutyric acid, as well as antiradical and antioxidant properties, thus effectively alleviating the anxiogenic behavior induced by sunset yellow in vivo [142].

### 6.19. Antivenom Activity

Suji et al. [143] demonstrated that the hemolysis caused by the venoms of *Daboia russelii* and *Naja naja* was alleviated by an aqueous root extract of *V. cinerea*. The hemolysis of the venoms was decreased to 50% and 40%, respectively, and the extract also decreased the hemolytic halo produced by the venom. Red blood cells were used to perform direct hemolysis tests using the venom samples of *N. naja* and *D. russelii*. The results indicated that both snake venoms could effectively lyse red blood cells, thereby producing total protein contents of 840 mg/mL and 222.2 mg/mL, respectively. In addition, phospholipase A_2_ assays demonstrated that 10 μg of the *D. russelii* and *N. naja* venoms could form hemolytic haloes measuring 10 mm and 11 mm, respectively, thus further demonstrating the antivenom capabilities of *V. cinerea*.

### 6.20. Toxicological Effect

In vivo experiments have indicated that mice and brine shrimp were not toxically affected by the methanol extract of *V. cinerea* [100]. In a mouse-based acute toxicity study, the median lethal dose (LD_50_) of the extract was above 2000 mg/kg without any pathological changes observed in a necropsy examination. In a brine shrimp lethality-based oral acute toxicity study, the LC_50_ values were 2.72 mg/mL at 24 h and 3.87 mg/mL at 6 h, thus indicating no noticeable toxicity.

Cyclophosphamide (CTX) is widely known as an antineoplastic drug, but its metabolites can cause toxicity in normal cells [144]. Pratheeshkumar et al. [97] demonstrated that the methanol extract of *V. cinerea* at doses of 10 mg/kg and 20 mg/kg, administered intraperitoneally, protected against CTX-induced toxicity in BALB/c mice. This was observed through an increase in total white blood cell count, bone marrow cellularity, *α*-esterase-positive cells, and lymphoid organ weights compared to the CTX group. Additionally, secondary metabolites isolated from *V. cinerea*, such as sesquiterpene lactones, reportedly exert no cytotoxicity on RAW264.7 cells in vitro, with cell viability between 97.6% and 114.3% [72].

## 7. Conclusions

*Vernonia cinerea* has long been acknowledged as a valuable nutritional resource and an essential ingredient in traditional medicine across many countries. It is known for its abundance of phytonutrients and diverse bioactive secondary metabolites, notably sesquiterpene lactones and various terpenoids, which contribute to *V. cinerea*‘s rich nutritional profile. Extracts from *V. cinerea* exhibit promising pharmacological properties, including antioxidant, anti-inflammatory, antitumor, and antidiabetic effects, and demonstrate efficacy in aiding smoking cessation efforts. Further investigation into the mechanisms of the bioactive compounds isolated from *V. cinerea* is necessary to elucidate its significant traditional medicinal applications and elucidate new ones. Moreover, these studies hold potential for the development of *V. cinerea* extracts and bioactive compounds as valuable sources for natural functional food ingredients and pharmaceutical therapies.

## Data Availability

Not applicable.
